# A comprehensive review of current knowledge on penile squamous cell carcinoma

**DOI:** 10.3389/fonc.2024.1375882

**Published:** 2024-05-22

**Authors:** Nishanth Thumma, Neharaj Pitla, Vasavi Gorantla, Maira du Plessis

**Affiliations:** Department of Anatomical Sciences, School of Medicine, St George's University, True Blue, Grenada

**Keywords:** penile cancer, penile squamous cell carcinoma, PSCC, penis, male reproductive cancers, penile intraepithelial neoplasia, PEIN

## Abstract

Neoplasm of the penis is relatively rare in most regions representing 0-2% of cancers worldwide. While the penis can be affected by sarcomas, basal cell carcinomas or even melanoma, Penile Squamous Cell Carcinoma (PSCC) represents approximately 95% of all penile neoplasms. Despite its rarity and most common presentation at later decades of life most individuals diagnosed with PSCC are faced with significant decrease in quality of life. The prevalence and incidence vary among different regions and populations, but a common trend is for diagnosis to occur late (stage 4). Underdeveloped countries are traditionally reported to have higher incidence rates; however, rates may vary significantly between urban and rural areas even in developed countries. Age adjusted rates are on the rise in some countries that used to have incidence rates of 1:100 000 or less. The list of associated risk factors is long and includes among others, lack of neonatal circumcision, poor genital hygiene, socioeconomic status, history of human papillomavirus (HPV) infection and penile intraepithelial neoplasia (PeIN). Many risk factors are widely debated among experts however HPV and PeIN are indisputable risk factors, and both also form part of the classification system for PSCC. Both conditions may have occurred in the past or be present at the time of diagnosis and identifying them plays a major role in management strategies. For such a rare condition PSCC can present in many different forms clinically making diagnosis no easy feat. Diagnosis of PSCC is done through clinical examination, including lymph node palpation, followed by a biopsy, which is essential for the classification. Lymph node involvement is a common finding at first presentation and investigation of spread to deep nodes is important and can be done with the aid of PET-CT. Treatment options for PSCC include surgery, chemotherapy, and radiation therapy. Surgical removal of the tumor is considered the most effective however can lead to severe decrease of quality of life. Chemotherapy is used in the case of fixed or bulky lymph nodes, where surgery is not indicated, and for distant metastasis. Radiation therapy is particularly effective in the case of HPV-positive PSCC.

## Introduction

Penile squamous cell carcinoma (PSCC) is a rare condition with significant variability in the distribution and reporting of incidence and prevalence worldwide. As a result of its rarity, not enough data exists to elucidate a clear pathogenesis, and it is considered to arise either as a malignant transformation of penile intraepithelial neoplasia (PeIN) or *de novo*, possibly in response to the presence of one or more risk factors (1). Significant challenges exist for conditions as rare as PSCC in finding effective treatments, especially in advanced stages (2). A significant concern with reporting cancer data, in general, is the misrepresentation and often interchangeable use of incidence and prevalence. This, combined with the scarcity of large cohort studies, creates difficulty discerning trends, causative and preventative factors, and population differences (2). While malignant tumors of the penis remain rare, the effects of the condition, especially with late-stage diagnosis, can be devastating, often leading to a severe decrease in quality of life (1–3). As with all cancers, treatment and prognosis depend heavily on how close to onset it is identified and how far it has spread (1, 4). The tumor’s size, location, and desire to maintain functionality determine the treatment plan. Surgical resection of the primary tumor remains the gold standard with the most promising prognosis but may not always allow for full organ functionality (1, 3, 4). The current consensus is that PSCC is the most common type of cancer found in the penis, and although it can be located anywhere on the organ, the glans and inner aspect of the prepuce are the most common sites of origin (2). Several risk factors have been suggested, including poor genital hygiene, lack of neonatal circumcision, HPV infection, and unsafe sexual practices (2). There is some discrepancy in reports regarding the risk of PSCC and socio-economic status, as well as the role that penile intraepithelial neoplasia (PeIN) plays in developing PSCC. These effects appear to be region or population-specific (3, 4). Over the past few decades, the incidence (the rate of occurrence over a time period expressed per 100000 individuals), prevalence (the total number of individuals with a condition in a set time period expressed as a percentage), and mortality rates have changed significantly (3). In addition, the risk factors and socio-economic status associated with it have also changed. It suggests that re-evaluation of what is currently known about both PSCC and the possibly associated PeIN, their risk factors, and associated treatments is timely. In this review, we aim to explore the anatomical considerations, reported risk and associated factors, and the change in the incidence of PSCC to paint a complete and current picture of this condition.

## Methods

Literature searches of databases including PUBMED, Google Scholar and Medline were done during 2022 and 2023 using the following keywords: Penile cancer, Penile squamous cell carcinoma and Penile intraepithelial neoplasia. Initial articles found were cross referenced for additionally relevant resources. After establishing the related factors and benign penile lesions that should be considered during deferential diagnosis, additional searches were performed for each condition and risk factor. The GLOBOCAN database was also consulted separately to determine the current incidence. Only articles published in English were included in this review, but no publication date restrictions were used.

## Review and discussion

### Anatomical considerations

Before discussing the incidence, epidemiology, and risk factors of penile squamous cell carcinoma, it is vital to understand the anatomical and histological structure of the penis and its surrounding tissue. The penis has three parts, the root, body, and glans, and consists structurally of paired erectile bodies (corpora cavernosa) that contain smooth muscle surrounded by several layers of fascia (5–7). Proximally the root is anchored to the perineal membrane and continue distally to form the body of the penis. The most distal portion is two separate rounded ends that terminate into the glans (7). Ventral to the two corpora cavernosa lies a third erectile body, the corpus spongiosum, containing within it the anterior urethra. It expands distally to form the glans surrounding the rounded ends of the corpora cavernosa. Several layers of fascia separate the erectile bodies from the overlying skin creating protective barriers between the different components (5, 6). The final and most superficial layer of the penile coverings is the skin, overlaying the highly vascular dartos fascia, the skin expands over the glans as a retractable double fold, the prepuce or “foreskin.” The histological structure of the skin of the penis is similar to that typically seen in thin skin; the prepuce and glans, however, have several unique characteristics with layers similar to that found in the gastrointestinal tract (8).

The prepuce lacks the dense collagen network typically associated with the dermis elsewhere in the body. The papillary layer of the dermis contains vast amounts of lymphoid cells, and a dense capillary network with sparce hair follicles and few sweat and sebaceous glands on the external surface (8). The presence of sebaceous glands on the inner (mucosal) surface of the prepuce has been debated, and the lack of evidence on humans suggests their absence (8–10). The density and arrangement of dartos muscle in the penis and prepuce specifically changes with age. Infants have a more mosaic-like muscle and elastin arrangement pattern, which is denser than that found in adults. It appears that this causes the prepuce to close the urethral orifice in infants, creating what can be described as a one-way valve system. This usually disappears in adulthood, leading to a more relaxed prepuce with more elastin than muscle being present (8). Due to the inner surface of the prepuce (that which faces the glans) consisting of mucosa rather than epithelium, a lamina propria layer is present deep underneath the dermis. It is highly vascularized and contains looser collagen than that found in the glans penis (8). The inner mucosal aspect of the prepuce is said to contain apocrine glands that secrete a myriad of protective cytokines and enzymes. Factors such as cathepsin B, lysozyme, chymotrypsin, neutrophil elastase, cytokine, and pheromones have been identified in the region. The bacterial flora of the preputal space is comparable to the eyes, mouth, skin, and female genital tract (9, 10).

The lymphatic drainage of the penis is an interconnected network of channels which may or may not converge or join from various regions (5). Generally, the skin of the penis and prepuce drain into one of the sets of superficial inguinal nodes initially. The erectile tissues and glans are commonly explained to drain into the deep inguinal nodes but may also drain into the femoral and superficial inguinal nodes (5–8). The lymphatics from the prepuce join the dorsal lymphatics from the body of the penis. Both together travel to the root of the penis and then diverge into the superficial inguinal lymph nodes. Lymphatics of the glans drain through 3 pathways: leading to the superficial inguinal, femoral or deep inguinal, or external iliac lymph nodes (5). The superficial inguinal lymph nodes are divided into the superolateral, superomedial, inferomedial, inferolateral, and central zones. The lymphatics from the penis mainly drain into the superomedial, central, or inferomedial zones. The sentinel node from the penis is generally found in the superomedial zone (5).

### Epidemiology

Penile cancer is often considered a disease of the elderly affecting mostly men between 50 and 70 years regardless of geographic location (1, 2, 11–14). It does, however, affect individuals of all ages with a range of 20 – 90 years and a mean age different for each country and region; it has also been reported in children (1–3, 11, 13, 14). Patient age has been associated with disease severity but not risk factor association. Younger individuals are reported to have minor lesions, and older individuals present more commonly with invasive disease (11). Despite the accepted mean age at diagnosis for PSCC being 60 years of age, approximately 19% of diagnosed patients reported are less than 40 years old (15, 16).

When reporting on the burden of specific cancers in different regions of the world, the factor of difference in age distribution should be eliminated, which is done by adjusting the crude rate by a standardized factor for each age group, which makes the incidence rates between regions more comparable and is termed age-standardized rate (ASR). In addition to the variance between countries, reports have shown that within countries, there is an uneven distribution of ASR between urban and rural areas. Generally, the incidence and prevalence of PSCC are lower in developed countries than in developing or underdeveloped countries (1, 2, 12, 13).

Reports on the incidence of penile cancer have varied significantly over the past 30 years and seem to be continually changing. According to the most recent (2020) GLOBOCAN data, the highest age-adjusted incidence rate of penile cancer is 7/100000 in Eswatini (former Swaziland), followed by Uganda and Botswana, with 4.6 and 4.4, respectively. The small island state of St Lucia is in fourth place with 3.9/100000; however, when looking at crude incidence rates, St Lucia has the highest incidence of all countries (17). The small population size with many elderly individuals accounts for the high incidence.

In 2003, cancer incidence in the United States and other developed countries (mainly Western Europe) was reported at or below 1/100000, contributing to only 1% of all reported cancers (11). By 2005, the number had increased to 1.5/100,000 in the US (18), subsequently decreasing to between 0.3 and 1.0/100,000 by 2015 (14, 19–21). Continual decrease brings the current incidence to 0.5/100,000 for the United States. The picture in Europe is different, with an apparent increase in incidence in countries such as Luxembourg (1.6/100,000), Norway (1.3/100,000), Denmark (1.2/100,000) and Germany (1.1/100,000) all reporting higher incidences than previously (20, 22).

Similarly, reports of PSCC contributing to up to 20% of cancers in developing and underdeveloped countries were common until early 2000 (11, 12), with incidence rates reported as high as 19/100000 in some (12). In developing and underdeveloped countries incidence has decreased over the past three decades but some fluctuations have been observed. Reports from India in 2012 show significant disparity in the incidence of PSCC in rural vs urban areas (0.7-2.3 in 100,000 and 3 in 100,000, respectively), raising concern for reporting combined data from countries with unequal wealth distribution (13, 16). According to Favorito et al. (16), the incidence rate in Brazil ranged between 2.9 and 6.8/100,000, a number which nearly halved by 2010 (15, 16, 21) and has since further reduced, standing currently at 1.3/100,000 (17). In 1993, PSCC was considered the most common urogenital cancer in many developing countries, including those in Africa, Asia, and South America (11, 12). The prevalence of PSCC in Puerto Rico was reported to be at 20% in 1993 (11); by 2004, the prevalence had significantly decreased to 10% in developing nations, with a minor 0.4-0.6% in the developed world, according to published data (2). Incidence data for PSCC for most developing and underdeveloped countries is not readily available, likely due to the lack of cancer registries in most of these regions until recently. However, a report by Chaux and Cubilla (20) showed the rates for South America, the incidence ranged between 4.2/100,000 in Paraguay, 1.5 – 3.7/100,000 in Brazil, 1.8/100,000 in both Columbia and Peru with the remaining regions ranging between 0.7 and 1.3/100,000 (20).

There is a great deal of emphasis in the literature on the difference in incidence rates between developed, developing, and underdeveloped countries, with the latter usually reported as having significantly higher incidence rates, which paints an unfortunate and inaccurate picture of the burden this condition poses (3). When considering the human development index and the incidence of PSCC reported in each country, there is a more even spread, with one of the most underdeveloped countries (Sierra Leone) reporting the lowest incidence of PSCC (0/100,000) (17). However, reports on the disparity between the stage at diagnosis and subsequent difficulty with treatment and increased mortality rate are not exaggerated. Several socio-economic and epidemiological factors have been suggested to play a role in the incidence and morbidity rates of PSCC.

### Pathogenesis

As stated previously, the pathogenesis of PSCC is not fully understood but appears to have a variety of possible associated factors. Evidence suggests that the development of PSCC may be multifactorial (3, 14). In addition, many conditions closely resemble SCC and should be considered as part of the differential diagnosis, including erythroplasia of Queyrat, Bowen’s disease, condyloma acuminata, and psoriasis, to name a few (1). Numerous factors have been associated with a higher prevalence of penile cancer, including poor hygiene, lack of circumcision, HPV infection, chronic inflammation, tobacco use, lichen sclerosus, history of phimosis, balanitis (inflammation of the glans), history of sexually transmitted diseases (STDs) particularly HIV, Penile intraepithelial neoplasia (PeIN) (1–3, 14, 15, 23), ultraviolet light exposure (24), and immune dysfunction whether caused by disease or medication (14, 22, 25). Other factors include mechanical injury or damage to the penile skin, chronic irritation (14, 15, 23), and history of genital warts (23). Although different studies show differences in correlation between PSCC and these factors, HPV infection, lack of neonatal circumcision, and poor genital hygiene are consistently quoted as significant risk factors (1). Moreover, HPV infection has been linked to the majority of PeIN cases (1), which in turn may be associated with approximately 50% of PSCC cases (1, 22).

Penile intraepithelial neoplasia (PeIN) is the term used for histological dysplastic changes of the penis without basement membrane infiltration (22, 25–29). It typically includes three conditions, which are all thought to be premalignant: erythroplasia of Queyrat (EQ), Bowen’s disease (BD), and Bowenoid papulosis (BP) (22, 25–29). All three of these conditions are said to be histologically similar to each other and to squamous cell carcinoma *in situ;* however, their gross morphology and clinical presentation vary considerably, making them distinct diagnoses (22, 29). Erythoplasia of Queyrat (EQ) is characterized by moist red plaques on the inner surface of the prepuce and mucosal surface of the glans; it may have one or more lesions present concurrently (29). Bowen’s disease (BD) is a single scaly lesion present on the keratinized area of the genital skin. Bowenoid papulosis (BP) is characterized by multiple small, well-demarcated papules with a pink, red, or brown color and can present on most areas of the penis (29). Other conditions can mimic BD and BP, such as lichen planus (LP), psoriasis, genital warts, and basal cell papilloma. In comparison, erosive LP and Zoon’s balanitis can present similar to EQ (29).

An important consideration is the histological similarity between BP and SCC *in situ*, suggesting they are the same (29); neither condition transforms into an invasive disease (29). PeIN may recur after treatment depending on the type, treatment modality, and the presence of risk factors (22, 29). EQ and BD are both considered to have malignant potential, which is exacerbated by a history of smoking, HPV infection (whether current or past), LP, and poor hygiene (29). Research has shown that the majority of PeIN is associated with high-risk HPV infection, which does not need to be present at the time of presentation (29). Additionally, the likelihood of an individual developing invasive PSCC increases with HPV associated EQ, BD and BP (3). More recent research has shown that PeIN can be subdivided into three distinct conditions, each associated with PSCC development and presentation (22). The classifications are differentiated, further divided into non-HPV-related and lichen sclerosus-related, and undifferentiated, which is also HPV associated (22). While these classifications are clear, the clinical picture is not always as clear-cut and should be considered when evaluating the patient (22). It is important to note that PeIN and PSCC may be present concurrently and that in the vast majority of cases, PeIN is associated with HPV infection (whether past or present) (22, 29). From published data, it can be seen that HPV infection is the most significant risk factor for the development of penile cancer, with nearly 50% of PSCC testing positive for HPV-16 or -18 (22, 29, 30). Studies have shown that tumors originating in the foreskin are different from those originating in the glans, suggesting that poorly differentiated HPV- related carcinomas are more likely to develop around the glans, where the mucosa of the urethral meatus merges with the glans (Central tumors) (20). Foreskin tumors are majorly HPV-unrelated and present as low-grade keratinizing carcinomas (peripheral tumors) (20).

It is considered that lack of neonatal circumcision is a strong risk factor for the development of PSCC; however, research has shown that not all individuals with PeIN are uncircumcised (22). When one also considers that more than half of PSCC occurs on the mucosal surface of the prepuce, it is easy to see a correlation (1, 22, 29, 30). It is important to note that research has shown that circumcision performed later in life, especially that associated with treatment for phimosis, carries an increased risk of developing PSCC (23). Therefore, while there is a higher prevalence of PSCC in un-circumcised vs circumcised individuals, other factors related to the presence of the prepuce should also be considered (22, 29, 30). In particular, Tseng et al. (23) demonstrated that the protective effect of neonatal circumcision is most likely due to the prevention of phimosis (18, 23).

Desquamated epithelial cells, mucin secretions from urethral glands of Littre, together with lipids, enzymes, and hormones, may form a white paste that accumulates between the glans penis and foreskin in uncircumcised males and is known as smegma (1, 9, 14, 31). It is essential to know that while smegma may be more substantial in uncircumcised males, it is also present in minor quantities in both circumcised males and females. The function of smegma is associated with lubrication of the thin mucosal lining of the inner prepuce and glans, allowing for smooth skin retraction during sexual activity (9, 10, 31). Studies have shown that in most individuals, it is an odorless substance that is usually colonized by several types of bacteria, even in infant boys (9, 32), but cannot be linked directly to an increased risk of penile cancer (10, 14, 31). Although some would argue that the presence of smegma alone is a risk factor for penile cancer, not enough evidence exists to show a direct correlation (9). Smegma can, however, become infected if the subpreputal region is not kept hygienic, transforming into inflammatory smegma. Inflammatory smegma can irritate the mucosal portions of the prepuce and glans and lead to dermatological changes, which have been established as a risk factor for PeIN and PSCC development (1, 4, 14, 22, 23). The presence of smegma has also been linked to phimosis, a non-retractable foreskin, which has been reported to increase the risk of developing penile cancer (4, 23).

While the correlation between phimosis and PeIN and PSCC seems to be clear (14, 15, 18, 23), studies have shown that in many cases, the presence of phimosis prevents the accumulation of smegma in the subpreputal space, thereby indicating that it is the non-retractable nature of the prepuce that leads to a higher risk of developing neoplasia (9, 31). The presence of phimosis can cause inflammation and injuries that facilitate HPV and other infections (14). The development of phimosis has also been linked to the presence of Lichen Sclerosus (LS) (14). Lichen sclerosus (LS) is a chronic inflammatory condition of the epithelium and lamina propria of the penis affecting the glans, foreskin, frenulum, urethral meatus, and anterior urethra. However, rare involvement of the penile body is known to cause hemorrhagic lesions (14, 33). It is characterized by atrophic and sclerotic plaques, which start as polygonal white raised patches (14). A study by Singh and Bunker (34) revealed that 50% of penile cancers were associated with LS, which may also be present in combination with HPV, leading to PeIN and PSCC (14, 34, 35).

Direct penile and glans or prepuce injury or tear are consistently associated with developing PSCC (14, 15, 18, 23, 36). The injuries may be as distant as two years before the presentation of penile cancer; even small tears have shown a significant risk of cancer development (15). PSCC has also been found on circumcision scars of adults who were circumcised to treat phimosis (22). History of penile rash and genital warts are also strongly associated with PSCC diagnosis (15, 18, 23, 36). Interestingly, Daling et al. (18) showed that circumcised individuals were significantly more likely to develop genital warts than uncircumcised individuals (18). Ultraviolet radiation is also identified as a significant risk factor for PSCC. A cohort study of 892 men revealed that men with prolonged exposure to PUVA during their psoriasis treatment are 300 times more at risk than others. However, local immunosuppression from PUVA and infection with HPV may have played a key role in causing these tumors (24).

### Diagnosis, classification, and staging

As previously mentioned, penile cancer may appear as a *de novo* lesion, a transformation of an existing precancerous lesion, or in response to treatment of dermatological conditions of the penile skin (1). Initial diagnosis is typically made based on clinical manifestations and lymph node palpation, followed by biopsy and imaging if diagnosis is uncertain (1, 37, 38). An investigation into the history of risk factors must accompany a typical investigation of the lesion. Careful attention to the characteristics of the lesion, such as diameter, location, number of lesions, morphology, and relationship to other structures, is necessary for accurate diagnosis (37, 38). Since histological and gross morphological classification of the tumor determines the evaluation of staging, treatment, and prognosis, careful evaluation is essential (28, 39–41).

#### Assessment of Lymph Node involvement

The lymphatic metastasis of PSCC is predictable, and in most instances, it predominantly drains into the superficial and deep inguinal lymph nodes first (1, 14, 38, 41, 42), depending on the site of the primary lesion. The spread may continue to the pelvic and the aortic nodes (1, 14). Lymph node palpation forms an essential part of examination at first presentation (1); however, most palpable nodes identified at this time are due to inflammation, not metastasis (38). If these nodes are not palpable, ultrasound may be used to identify any nodes and to guide fine-needle aspiration for investigation of malignancy (38, 41). MRI and PET-CT have been shown as reliable imaging modalities to aid in identifying malignant inguinal lymph nodes (38, 42). Although distant lymph node involvement is rare (1-10%), an investigation of distant lymph nodes, such as the pelvic and abdominal lymph nodes, should be done if the inguinal lymph nodes are positive for metastasis. PET-CT is a reliable method of assessing distant lymph nodes for metastasis (1, 14, 38, 43). Guidelines by the National Comprehensive Cancer Network have specific recommendations regarding the need for CT evaluation of the body cavities for each stage. For cancers staged T1b and grater CT of the chest, abdomen and pelvis is recommended, regardless of palpable lymph nodes. This should be accompanied by, at the very least, bilateral sentinel node biopsy (44). This is not surprising as research has shown that between 11 and 60% of lymph node metastasis is missed on initial screening (7) since micro metastasis occurs frequently and does not enlarge the node substantially enough to be detected on palpation (7).

#### Classification and histological grading

The classification of PSCC is no simple task, and is based on histological and molecular genetic characteristics, pathogenesis, and prognosis. One aspect that complicates the histological grading of these cancers is the common co-occurrence of PSCC and PeIN (45). A separate grading and classification system exists for PeIN but histological grading of PSCC may include PeIN as part of the grade (45). Two main subclassifications are used because of the difference in cause, appearance, progression and prognosis: HPV-associated and non-HPV-associated (21, 28, 39, 45). **Table 1** shows the most current (and common) classification system used as described by the Union Internationale Contre le Cancer (UICC), AJCC 8th ed, 2022 and NCCN 1.2024 (20, 21, 44–46), due to the rarity of some classification descriptions are not always available. It should also be noted that currently PeIN and Carcinoma *in situ* are considered as synonymous (44, 45).

**Table 1:** PSCC classification based on UICC guidelines, modified from Chaux and Cubilla (2012) (20), Hakenberg et al. (2018) (21), NCCN 1.2024 (44) and Sanchez et al., 2022 (45).

Classification	Approximate frequency	Macroscopic appearance	Microscopic appearance
Non-HPV-associated			
Squamous cell carcinoma, common type	70-75%	Appear as slightly raised areas of skin	May be associated with or without keratinization
Pseudohyperplastic carcinoma	<1	Multicentric, one of the tumors may present with verrucous or papillary features	Low-grade histology that simulates pseudoepitheliomatous hyperplasia
Pseudoglandular carcinoma	<1	Tumors may have a honeycomb appearance, simulating glandular tumors with acantholytic or adenoid features because of central necrosis in tumor nests	High-grade and highly metastatic
Verrucous carcinoma	2-3%	Exophytic, verrucoid lesions with a characteristic cut surface: white, superficial, and with a broad base separating the tumor from the stroma	Papillomatous, acanthotic and extremely well-differentiated
Carcinoma cuniculatum	<1	Variant of verrucous characterized by a distinctive labyrinthine growth pattern with tumor tracts and sinuses, often with foreskin fistula formation	Similar to hybrid verrucous carcinoma
Papillary carcinoma, NOS	5-8%	Exophytic, large tumor on the cut surface. shows a jagged interface between the tumor and the stroma	Papillomatosis and low-grade histology. No koilocytosis
Adenosquamous carcinoma	1-2%	Mixed tumor characterized by predominant squamous tumor nests intermingled with focal areas of glandular differentiation	High grade, with vascularization. Glandular component positive for mucin stains and CEA
Sarcomatoid carcinoma	1-4%	Macroscopically, may be polypoid or highly hemorrhagic and necrotic	Simulate various sarcomas, such as malignant fibrous histiocytoma, leiomyosarcoma, fibrosarcoma, or osteosarcoma
Basaloid carcinoma	5-10%	Ulcerative, nonspecific aspect. cut surface is typical with a solid, tan, firm, moderately to deeply invasive neoplasm with minute yellow foci of necrosis	Nesting pattern, often confluent, each nest conforming to the histologic landmark: solid or centrally necrotic. Peripheral palisading may occur. Cells are small or intermediate, basophilic, basaloid, spindle, or pleomorphic, with many apoptotic and mitotic figures
Papillary basaloid carcinoma	rare		Papillae lined by small basophilic cells
Warty carcinoma	5-10%	Verruciform tumors with a cobblestone appearance. Cut surface shows an exoendophytic tumor with jagged interface between the tumor and the stroma	Condylomatous papilla lined by clear cells. Central fibrovascular core and keratinized cells with superficial and deep pleomorphic koilocytosis
Warty basaloid carcinoma	rare	Distinctive heterogeneous neoplasms that, on macroscopic examination and cut surface, reveal a biphasic exoendophytic invasive tumor	Mixed features of warty and basaloid carcinomas are noted. Frequently, a papillomatous tumor is observed at surface, with an underlying basaloid carcinoma and central clear cells invading corpus spongiosum or cavernosum
Clear-cell carcinoma	rare	Large and sharply demarcated, sometimes ulcerated, measuring 2 to 5 cm	Highly invasive tumor nests composed of clear cells that are large with well-defined polygonal shape
Lymphoepithelioma-like carcinoma	rare		
Medullary carcinoma	rare		

Other factors taken into consideration when characterizing PSCC’s growth pattern. The classifications described in **Table 1**, and the growth patterns described below provide a better description of the individual case at hand and a better prediction for prognosis (28, 39). Though 33% of the penile tumors show a mixed growth pattern, there are five principal growth patterns (39).

a. Superficial spreading.
b. Vertical growth pattern.
c. Verrucous.
d. Multicentric.
e. Mixed.

Superficial spreading is the most common growth pattern observed in carcinomas and involves superficial anatomical layers of the glans, foreskin, and sulcus. This growth pattern is usually associated with the usual type of non-HPV-related SCC (39). It has different presentations that consist of: a) primarily in-situ carcinoma, with or without superficial infiltration into lamina propria; b) focal vertical pattern of growth (nodular and deeply invasive), which is predominantly in-situ carcinoma; c) horizontal and band-like growth of carcinoma and entirely comprised of a mix of in-situ and superficially invasive carcinoma (39). The gross appearance of the vertical growth pattern shows a large, ulcerated, fungating mass on cut sections. This growth pattern is associated with the histological subtypes basaloid, high-grade usual SCC, and sarcomatoid (28). Almost 25% of PSCC are verruciform, showing well-differentiated hyperkeratosis with papillary configuration under a microscope. Verrucous, warty, papillary, and cuniculatum carcinomas show verruciform patterns of growth (28, 39). Pseudo hyperplastic carcinomas have a multicentric growth pattern, whereas mixed patterns present as a combination of superficial spreading, vertical, verruciform, and sometimes multicentric growth patterns.

Despite the consensus that grading plays an important role in prognosis and treatment there still appears to be differing opinion on how to approach the grading of PSCC. One of the problems with establishing a grading scheme is the lack of reproducibility of grading models by pathologists (45). Grading schemes range from 2-tier to 4-tier systems and many authors propose their own grading scheme based on clinical experience. The AJCC recommendation is for a 3-tiered system with grade 1: well differentiated, grade 2; moderately differentiated and grade 3; poorly or undifferentiated (44, 46). Sanchez et al., proposes the use of this same system applying rigorous scrutiny over grades 1 and 3 and awarding everything in between as grade 2 (45).

Once the classification of the lesion has been established, the stage needs to be determined. Staging of penile SCC is usually done based on the tumor both clinically and histologically by grade, regional lymph node, and distant metastasis (TNM) method as reported in the American Joint Committee on Cancer (AJCC 8^th^ ed) (46, 47). **Tables 2**–**5** show the elements considered during staging. It seems that developing countries more often have later stages of diagnosis, but this also appears to be the case in lower socio-economic areas of developed countries. Delayed detection of penile cancer is a result of multiple factors that may be linked to the possible risk factors. Embarrassment, guilt, fear, personal neglect, socio-economic status, and access to specialized services are some of the factors contributing to delayed diagnosis of penile cancer (16). Many cancers in developing countries are diagnosed at stage 3 or beyond, meaning that they have already metastasized into the regional lymph nodes, resulting in a poorer prognosis (4). The treatment plan is determined by the size and location of the tumor as well as the desire for functionality. Regardless, surgical resection of the primary tumor remains the gold standard with the most promising prognosis but may not always allow for full organ functionality (1, 4).

**Table 2:** Evaluation of primary tumor stage [as stated in the 7^th^ ed of the AJCC Cancer Staging Manual (46), NCCN 1.2024 (44) and Sanchez et al., 2022 (45)].

Primary Tumor (T)	
TX	Tumor cannot be assessed
T0	No evidence of tumor
Tis	Carcinoma *in situ* (also referred to as Penile intraepithelial neoplasia (PeIN))
Ta	Non-invasive localized SCC (broad penetration permitted)
T1	Invasive into underlying tissue unique to region: Glans: invades into lamina propria Prepuce: invades into lamina propria, dermis or dartos fascia Shaft: invades underlying connective tissue but not corpora No associated lymphovascular or perineural invasion
T1a	Tumor invades subepithelial connective tissue, no neural, lymphatic or lymphatic vascular involvement and not poorly differentiated
T1b	Tumor invades subepithelial connective tissue with neural and/or lymphatic or lymphatic vascular involvement or is poorly differentiated
T2	Tumor invades corpus spongiosum with or without involvement of the urethra
T3	Tumor invades corpus cavernosum with or without involvement of the urethra
T4	Tumor invades other adjacent structures including prostate, pubic bone or scrotum etc.

**Table 3:** Evaluation of lymph node involvement stage [as stated in the AJCC Cancer Staging Manual, 7^th^ ed (46), NCCN 1.2024 (44) and Sanchez et al., 2022 (45)].

Regional Lymph Node (N) involvement			
Pathological		Clinical	
pNX	Regional lymph nodes cannot be assessed	cNX	Regional lymph nodes cannot be assessed
pN0	No regional lymph node metastasis	cN0	No palpable or visibly large inguinal lymph nodes
pN1	Metastasis in a single superficial inguinal lymph node	cN1	Unilateral mobile palpable inguinal lymph nodes
pN2	Metastasis in multiple or bilateral superficial inguinal lymph nodes	cN2	≥ 2 unilateral mobile palpable or bilateral palpable inguinal lymph nodes
pN3	Metastasis in unilateral or bilateral deep inguinal lymph nodes	cN3	Uni- or bilateral palpable fixed inguinal mass or pelvic lymphadenopathy

**Table 4:** Evaluation of distant metastasis stage [as stated the AJCC Cancer Staging Manual, 7^th^ ed (46), NCCN 1.2024 (44) and Sanchez et al., 2022 (45)].

Distant Metastasis (M)	
MX	Distant metastasis cannot be assessed
M0	No distant metastasis
M1	Distant metastasis

**Table 5:** Histological grading [as stated in the AJCC Cancer Staging Manual, 7^th^ ed (46), NCCN 1.2024 (44) and Sanchez et al., 2022 (45)].

Histological Grading (G)	
GX	Grade cannot be assessed
G1	Well-differentiated
G2	Moderately differentiated
G3	Poorly or undifferentiated differentiated

### Current management strategies

#### Penile preserving treatments

For many cancers an aggressive approach may be taken to ensure complete cure and survival of the patient. In PSCC, however, this practice has significant detrimental effects on the patient’s psychosocial health (1–3). Penile preserving procedures generally lead to a better Quality of life for the patient as compared to partial or radical penectomy (21, 48). PSCC which is limited to the foreskin can be effectively treated with circumcision however additional therapies may be required if there is lymph node involvement (21). For Tis or Ta, topical chemotherapy agents such as 5-Fluorouracil or imiquimod, both of which may be applied as aqueous solution, has been reported as successful. Other options such as wide local excision, complete or partial glansectomy, or Mohs micrographic surgery (MMS) may also be used for T1 and below all of which are current recommendations by NCCN (21, 44). For T1 lesions below grade 3 or Tis and T1 lesions persisting or recurring after topical treatments, laser ablation treatment have been shown to be effective (21, 44).

Wide local excision involves removing the tumor tissue along with unaffected tissue around it and is indicated for Tis, Ta and T1 with little or no invasion. Traditionally, a 10 mm clearance area of unaffected tissue was excised in cases of low grade tumors (grade 1-2), and 15 mm clearance area for higher grade tumors (grade 2 and above) (44, 49). However, research also suggests that smaller excision margins can be successfully used and is determined by the grade of the tumor (21, 50) glans resurfacing (removal of glans epithelium) may be also be considered in some cases (21, 44). Wide local excision is not performed in cases where the tumor is close to the urethra, where the tumor involves the meatus, or when the tumor extends more than 50% of the glans penis (49). Partial or complete glansectomy can be used for Tis, T or T1 tumors that involve only the glans and/or prepuce but are not indicated for G1-2 T1 tumors (44). Some authors report success with glansectomy in T2 tumors of the glans but this is not part of the current NCCN recommendations and may be due to the lack of sufficient evidence on this rare topic (21). For glansectomy cosmetic repair is possible with skin grafts for some patients however it must be considered that sensory innervation to the area will be completely lost (21).

Mohs micrographic surgery is an effective treatment option for individuals with penile squamous cell carcinoma, providing a tissue-conservative alternative to partial or total penectomy. The procedure involves sequential tissue excision while viewing under a microscope (51). The sections are mapped and repeated until no more tumor cells are identified. It has shown remarkable efficacy, demonstrating high cure rates and reduces local recurrence while ensuring maximal organ preservation. Notably, a comprehensive 30-year retrospective study of 42 patients showed a remarkable cure rate of 94.7% for primary SCCs *in situ* and an initial cure rate of 66.7% for recurrent invasive SCCs, with successful re-treatment in recurrent cases (52). Furthermore, a comprehensive analysis of MMS for penile cancer cases between 1988 and 2006 highlighted its effectiveness despite a high local recurrence rate (51). The study, which included 33 patients and 41 procedures, emphasized that diligent follow-up and repeat procedures contribute to excellent cancer-specific and overall survival rates with low progression rates. This evidence demonstrates that MMS is a valuable and adaptable treatment approach for penile SCC.

Local recurrence is an important factor to consider when planning treatment, laser ablation has been shown to have a 10-48% recurrence rate with glans resurfacing and glansectomy following at 0-6% and 0-2% respectively (21). This however is not a concern as local recurrence can simply be treated with the same measures as before and preserves maximum functionality of the organ (21).

#### Radiation and chemotherapy

Radiation is generally effective for squamous cell carcinomas and can be used alone or in conjunction with other organ sparing techniques (21, 53) and has also been shown to be beneficial for HPV-positive penile cancer (4). Brachytherapy (BT), a form of internally administered radiotherapy has been shown to be successful in early-stage cancer (T1-2) with no nodal involvement, when used in combination with circumcision when only the glans is involved (54). It maintains full functionality of the organ and has excellent long-term control as shown by de Crevoisier et al. (54). External beam radiation therapy (EBRT) is indicated for T1-2 tumors without nodal involvement in tumors of less than 4cm following organ sparing surgical removal and may be used with or without combination with chemotherapy. For patients in whom surgery is not indicated or those who may refuse surgery EBRT of the inguinal region may be used as a preventative measure against nodal spread (44). For T1-2 tumors without nodal spread that are larger than 4cm EBRT in combination with chemotherapy is indicated after circumcision, in these cases BT may be used alone but post-therapy surveillance is required (44).

For T3 and above with nodal involvement that is unresectable the entire penile shaft and bilateral inguinal region as well as pelvic nodes are treated with EBRT and concurrent chemotherapy after circumcision (44, 53). The lesion itself with a 2cm margin and inguinal regions are then treated with additional EBRT at a higher radiation level. Surveillance and follow-up treatments must be considered in all surgical removals especially at the surgical margins. If tumor formation is observed at the surgical margin NCCN recommends treatment with EBRT at surgical site and scar region, if lymph node dissection was not done at time of surgery inguinal region EBRT is also indicated (44).

Adjuvant radio- or chemotherapy may also improve outcome in patients with distant metastasis but causes side effects such as severe edema and pain. It is recommended in patients with positive inguinal and/or pelvic lymph nodes especially if they are bulky, bilateral or fixed to the underlying tissue (37, 38, 44). The NCCN guidelines also recommend radiation therapy as part of palliative care to be delivered in 10 fractions. Adjuvant chemotherapy is given to patients with positive inguinal lymph nodes larger that 4cm prior to surgical resection. The chemotherapy may also be given post-surgery either should the need be there and is indicated in cases where post-resection pathology shows high risk features (44). However, the NCCN guidelines highlight that there is insufficient data to make definitive recommendations.

Surgery for fixed or bulky lymph nodes (N3) is not recommended due to a decreased likelihood of cure. In this case, neoadjuvant chemotherapy is recommended. Paclitaxel, cisplatin, and ifosfamide (TIP) are examples of drugs used in neoadjuvant chemotherapy regimens (36). This same chemotherapy regiment is used in pre- and post-surgical chemotherapy for radical lymph node resection There are no guidelines regarding adjuvant therapy for penile SCC. However, based on the neoadjuvant chemotherapy data, it may be reasonable to administer TIP as adjuvant therapy for patients with high-risk features such as palpable inguinal lymph nodes and pelvic lymph node involvement (37). For metastatic PSCC, either TIP or a combination of 5-fluorouracil and cisplatin can be used as first-line therapy (37).

#### Non-penile sparing treatments

Surgical removal of all or part of the penis is considered the best treatment option for most penile tumors above T3 and those with high grade (4, 21). The extent of removal and required adjuvant therapy is based on the grade and stage of the tumor as well as the possibility of ensuring an adequate surgical margin. NCCN guidelines recommend that if a suitable margin cannot be obtained when attempting to leave a functional stump total penectomy should be performed (44). In cases of inguinal lymph node metastasis, inguinal lymph node dissection (ILND) is usually recommended (37). Traditionally, radical inguinal lymphadenectomy (radical ILND) is performed, in which the long saphenous vein is cut at the top of the femoral triangle. Lymph nodes anterior to the femoral artery and vein are dissected, and the sartorius muscle is transposed to cover the femoral vessels (48). Radical ILND was associated with considerable morbidity due to common complications such as wound infections, necrosis, and venous thromboembolism (48). Modified lymphadenectomy, pioneered by Catalona in 1988 (48), can decrease morbidity. This method uses a smaller incision, preserves the saphenous vein, and leaves the sartorius muscle in place (37). More recent studies have indicated the protective factor of early lymph node resection using modified techniques to improve success (7). This recommendation was made due to the frequent presence of micrometastases that were not detectable via conventional means (7). In the case of distant metastasis, such as metastasis in the pelvic lymph nodes, a pelvic lymph node dissection (PLND) can be done. However, this is usually only done in patients with two or more positive inguinal lymph nodes (ILN) or poorly differentiated metastases (37).

#### HPV vaccination

A significant number of cases of penile cancer are linked to HPV infection. Over 50% of penile cancer cases and almost 80% of PeIN cases are associated with HPV DNA (55). This data shows that HPV vaccination could help prevent penile cancer. A quadrivalent HPV vaccine was approved by the Food and Drug Administration in 2009. However, not enough studies have been done to prove that HPV vaccines directly prevent penile cancer. Hence, the use of HPV vaccines as a prevention or treatment method is not widespread (55).

### Sexual outcomes after treatment

Sexual health forms an important aspect of overall health and wellness. It consists of several aspects including, but not limited to, ability to obtain and maintain erection, achievement of orgasm, sexual desire, intercourse satisfaction and overall sexual satisfaction (56). Penile cancer and its treatment have a significant impact on sexual wellbeing, both physiologically and psychologically, with more than 40% of patients impacted negatively post-surgery in at least one of these domains (56–58). Organ sparing procedures are often considered to have better outcomes than more radical approaches (21, 48, 57) however, some reports have shown that sexual outcomes after WLE and glansectomy with urethral glanduloplasty still have some reduction in sexual function. Glanduloplasty patients appear to exhibit a worse international index of Erectile Function (IIEF-5) and Changes in Sexual function (CSFQ) scores post-surgery (57). This is likely due to the loss of sensory innervation to the glans due to the surgical procedure (21). Reports on erectile function after partial penectomy vary, with some studies reporting a significant reduction, others minimal reduction and one study an increase post partial penectomy (56). Some patients also report having no penetrative intercourse after surgical treatment, however it is unclear if this is by choice or due to functional loss. Whyte et al., reports in a systematic review that the majority of patients experience a decrease in ejaculation and orgasm post partial penectomy as compared to before the procedure. Likewise for sexual desire a general consensus is that patients experience a decrease post partial penectomy while some reports of an increase has been noted (56). A similar trend was observed for total intercourse satisfaction, this is linked to both frequency of intercourse and enjoyability thereof. According to available data is seems that the impact on sexual health is dependent both on patient demographics and the treatment method but also the psychological health of the patient (56–58). Factors such as age, partnership relationship and penile length post surgery seems to play a major role in reports of dysfunction and dissatisfaction with sexual activity. Older men in mature relationships reported strengthening of their relationship as intimacy, care and companionship became more important than penetrative intercourse (58). However, this is not the case for all. Younger patients tend to experience more devastating effects on sexual function when compared to older patients undergoing the same treatment procedure (57, 58). According to Cilio et al., the existence of other health conditions such as diabetes increases the risk of sexual dysfunction post treatment regardless of the nature of the treatment. Organ sparing treatments provide a significant advantage over less conservative treatment methods because of the maintenance of sexual function and associated increase in quality of life (57). It is important to note however that all reports emphasize the importance of pre- treatment patient education, this is especially important in organ sparing treatments as recurrence is fairly common. As noted previously, recurrence of penile cancer treated with conservative organ sparing means can simply be treated by repeat measures and are not associated with a significant change in survival (21, 57).

## Conclusion

In conclusion, penile squamous cell carcinoma is a rare and aggressive form of cancer that presents significant challenges in terms of diagnosis and treatment. Various treatment options, including surgery, radiation, and chemotherapy, are currently used palliatively to manage the disease, but there is limited potential for cure or survival benefit. The role of HPV in disease prognosis and treatment has recently come into focus, with studies showing its association with penile cancer and the potential for targeted therapies. It is crucial to consider the risk factors for penile squamous cell carcinoma, such as poor hygiene, smoking, lack of circumcision, and HPV infection, to reduce the risk of developing this type of cancer. While HPV vaccination has proven to be highly effective in preventing HPV-related vulval neoplasia, it, unfortunately, does not offer the same level of protection against penile cancers. Therefore, preventive strategies such as circumcision, avoidance of smoking, and practicing safe sex are crucial in reducing the risk of penile squamous cell carcinoma. Furthermore, managing advanced cases of penile squamous cell carcinoma requires a multimodal approach. Surgery remains the mainstay of treatment, but its potential morbidity and impact on quality of life can be minimized by performing penile-sparing surgery in early disease. The benefits for sexual health and associated increased quality of life associated with penile sparing techniques outweigh the possibility of recurrence.

## Author contributions

NT: Writing – original draft, Writing – review & editing, Investigation. NP: Writing – review & editing, Writing – original draft, Investigation. VG: Supervision, Writing – original draft, Writing – review & editing, Conceptualization, Methodology. Md: Investigation, Supervision, Validation, Writing – original draft, Writing – review & editing.
